# Six-month outcomes of a three-arm prospective study comparing Da Vinci vs. Hugo RAS vs. versius robotic radical prostatectomy: (the COMPAR-P trial)

**DOI:** 10.1007/s11701-026-03260-5

**Published:** 2026-03-19

**Authors:** Alessandro Antonelli, Alessandro Veccia, Sarah Malandra, Vincenzo De Marco, Riccardo Rizzetto, Alessandra Gozzo, Alberto Bianchi, Matteo Brunelli, Marianna Noale, Maria Angela Cerruto, Riccardo Bertolo, University of Verona Residency Program in Urology, Mariana Finocchiaro, Luca Rahmati, Mattia Ronca, Michele Aloe, Peres Fokana Pongmoni, Andrea Franceschini, Antonio Raiti, Endri Toska, Vincenzo Vetro, Francesco Artoni, Alberto Baielli, Claudio Brancelli, Sonia Costantino, Piero Fracasso, Francesca Fumanelli, Francesca Montanaro, Iolanda Palumbo, Greta Pattenuzzo, Luca Roggero, Michele Boldini, Davide Brusa, Giovanni Corghi, Lorenzo De Bon, Francesco Ditonno, Lorenzo Pierangelo Treccani

**Affiliations:** 1 Department of Surgery, Dentistry, Pediatrics and Gynecology, Urology Unit, University of Verona, Verona, Italy; 2 Residency Program in Health Statistics and Biometrics, University of Verona, Verona, Italy; 3 Department of Diagnostic and Public Health, Section of Pathology, University of Verona, Verona, Italy; 4 National Research Council (CNR), Neuroscience Institute IT, Pisa, Italy; 5 Azienda Ospedaliera Universitaria Integrata Verona, AUOI Verona, Borgo Trento Hospital, Piazzale A. Stefani 1, Verona, 37126 Italy

**Keywords:** DaVinci, Hugo RAS, Versius, Radical prostatectomy, Robotic surgery

## Abstract

To compare postoperative, oncological, and patient-reported outcomes of robot-assisted radical prostatectomy (RARP) performed with three CE-marked robotic platforms: Da Vinci Xi, Hugo RAS, and Versius. The COMPAR-P trial is a prospective, monocentric, post-market study conducted at the Azienda Ospedaliera Universitaria Integrata of Verona, Italy (ClinicalTrials.gov NCT05766163). From March 2023, 150 patients with organ-confined prostate cancer were consecutively enrolled and allocated to undergo RARP with Da Vinci Xi (n = 50), Hugo RAS (n = 50), or Versius (n = 50). Two high-volume robotic surgeons performed all procedures, experienced with Da Vinci but naïve to Hugo RAS and Versius before trial initiation. Surgical technique, perioperative protocols, and follow-up were standardized across cohorts. Outcomes at 6 months included serum PSA, complications (Clavien–Dindo classification), late sequelae (> 90 days), and health-related quality of life (SF-36 and UCLA-PCI questionnaires). Longitudinal analyses used linear mixed-effects models. Baseline demographics and disease characteristics were comparable across groups. At 6 months, PSA was undetectable in most patients, with no significant intergroup differences. Complication rates and late sequelae were low and evenly distributed. Questionnaire completion exceeded 90% at all time points. No significant long-term differences emerged in most SF-36 and UCLA-PCI domains. However, at 1 month, Hugo RAS and Versius patients reported lower Sexual Function scores than Da Vinci (–20 and − 28 points, respectively), and Versius patients reported lower Sexual Bother scores (–25 points, all p < 0.05). These differences disappeared at 3 and 6 months. Hugo RAS patients showed a temporary advantage in Physical Functioning at 1 month (+ 17 points vs. Da Vinci). Within the present study, and notwithstanding its inherent limitations, Da Vinci, Hugo RAS, and Versius achieved comparable safety, oncological, and short-term patient-reported outcomes in RARP. Early functional differences were transient and resolved over time. Surgical expertise, rather than the platform type, appears to be the primary determinant of outcomes.

**Supplementary Information:**

The online version contains supplementary material available at 10.1007/s11701-026-03260-5.

## Introduction

In the past few years, novel robotic platforms have been introduced, potentially increasing competition in the market, lowering the cost of robotics, and potentially expanding the number of patients benefiting from surgery performed via a robotic approach [1].

The Da Vinci Surgical System (Intuitive Surgical, Sunnyvale, CA, USA), has long been the gold standard in robotic-assisted surgery, dominating the field with its surgeon-friendly interface. Since its introduction, it has become synonymous with minimally invasive prostatectomy, offering three-dimensional visualization, enhanced dexterity with wristed instruments, and ergonomic control for surgeons.

The Versius (CMR Surgical Ltd, Cambridge, UK) and the Hugo RAS (Medtronic, Minneapolis, MN, USA) systems have emerged as other frontrunners in robotic prostatectomy after receiving the CE mark approval in 2021 and 2019. These platforms introduced novel features such as modular robotic arms and an open console architecture, promising to address eventual limitations associated with Da Vinci [2–4].

These systems have had a patchy penetration among institutions, so a few publications have appeared, generally represented by retrospective single-arm non-comparative studies [5].

There is an imperative need for a comprehensive comparative analysis between the two robotic platforms. Understanding the nuanced differences in efficacy, ergonomics, cost-effectiveness, and learning curves is essential for surgeons, healthcare institutions, and patients.

Accordingly, we promoted a prospective study to compare three platforms CE-marked for RARP head-to-head. The present paper reports granular data on patients enrolled in the three arms: Da Vinci versus Hugo RAS versus Versius RARP, to compare postoperative and patient-reported outcomes of RARP performed with these three platforms.

## Materials and methods

The COMPAR-P trial (Comparison of Outcomes of Multiple Platforms for Assisted Robotic Surgery - Prostate) is a monocentric, post-market investigation promoted by the Azienda Ospedaliera Universitaria Integrata (A.O.U.I.) of Verona, Italy [6]. It secured approval from the local ethical committee (4038CESC) and underwent registration on ClinicalTrials.gov (NCT05766163). The study was performed in accordance with the Declaration of Helsinki.

Enrollment commenced in March 2023. Patients diagnosed with organ-confined prostate cancer and deemed suitable for RARP at our department were assigned to one of the evaluated platforms until cohorts of 50 consecutive cases were completed. Every patient wishing to participate in the study provided signed informed consent.

The operating room personnel underwent intensive 3-day training at the ORSI Academy in Melle, Belgium. Two console surgeons (AA and VD), with prior experience exceeding 1000 and 500 da Vinci RARP interventions, respectively, conducted all the procedures. Importantly, none had performed any clinical procedure with the Hugo RAS or the Versius platforms before recruitment for this study started. Three additional surgeons, well-versed in robotic assistance (RB, RR, and AV), participated as table assistants. The anesthesiologic protocol and patient positioning (supine position, legs together and extended, 25° Trendelenburg) remained consistent regardless of the platform used.

Specific adaptations in port placement relative to the Da Vinci system were applied according to the platform used, as previously described [7, 8]. Consistency was maintained across platforms concerning insufflation and aspiration systems, robotic instruments (monopolar scissor, Maryland bipolar forceps, fenestrated grasp, and needle-driver), assistant instruments, sutures, and clips. The surgical technique was reproduced identically, regardless of the platform used.

The nerve-sparing technique was performed according to preoperative oncological risk based on clinical data and intraoperative findings. When indicated, an intrafascial or interfascial antegrade dissection was performed; otherwise, an extrafascial dissection was adopted.

After transecting the Santorini plexus, apical dissection was performed in an anatomical fashion, aiming to maximize urethral length whenever oncologically appropriate [9, 10]. The Santorini plexus was then selectively ligated [11]. Posterior reconstruction was performed using a running suture to restore the continuity of the posterior fascial tissues, whereas anterior reconstruction aimed to re-establish the continuity of the vesical apron with the puboprostatic ligaments [12].

The decision to perform a lymph-node dissection (LND) was based on the calculated risk of lymph-nodal invasion, determined by nomograms, and followed an extended template [13]. The postoperative course was managed according to the Institutional standard of practice in all cases.

The present study aimed to evaluate, with a 6-month follow-up, the differences among da Vinci vs. Hugo RAS vs. Versius in terms of:

1. serum prostate-specific antigen (PSA, considered undetectable when < 0.1 ng/mL);
2. postoperative complications (as classified according to Clavien-Dindo grade, considered as major complications if grade > 3);
3. postoperative sequelae occurring beyond 90 days (monitored up to 180 days postoperatively);
4. patients’ health-related quality of life (assessed by administering the Italian-validated versions of the SF-36 and University of California Los Angeles Prostate Cancer Index [UCLA-PCI] questionnaires [14, 15]).

De-identified data were systematically collected in a Research Electronic Data Capture (REDCap) dataset throughout the surgical procedures by an assigned investigator who did not participate in the surgeries. Access to REDCap was restricted to a specific password-coded computer securely stored within a locker. The password was changed periodically and managed exclusively by the involved investigators.

### Statistical analysis

All statistical analyses were conducted in accordance with international guidelines for clinical research reporting [16]. The sample size was determined based on study feasibility, resulting in 50 patients per cohort. The distribution of continuous variables was assessed using the Shapiro–Wilk test and visual inspection (histograms and Q–Q plots). Normally distributed data were summarized as mean ± standard deviation (SD), whereas non-normally distributed variables were presented as median and interquartile range (IQR, 25th − 75th percentile). Categorical variables were described as counts and percentages. Between-group comparisons were performed using the Student’s t-test for normally distributed continuous variables and the Mann–Whitney U test for nonparametric variables. Differences in categorical variables were analyzed using Pearson’s chi-square test or Fisher’s exact test when expected counts were < 5.

Longitudinal outcomes (SF-36 and UCLA-PCI scores) were analyzed using linear mixed-effects models with random intercepts for patients. Fixed effects included robotic platform, time, and the platform × time interaction. Adjustment variables were age at surgery, baseline questionnaire scores, preoperative serum PSA levels, and prostate volume.

Missing data were assumed to be random, and no imputation methods were adopted. The domain scores were calculated excluding the missing items. The domain score was not calculated if more than 50% of the items were missing. All tests were two-sided, with statistical significance set at *p* < 0.05. Analyses were performed using Stata version 18.0 (StataCorp LLC, College Station, TX, USA). Key commands included: swilk, histogram, ttest, tabstat, ranksum, tabulate, chi2, mixed, contrast, and margins.

## Results

During the study period, 150 patients were operated on, specifically 50 patients for each platform under investigation. The operated patients’ baseline demographic and disease characteristics were not statistically different when comparing platform versus platform, respectively [7, 8]. The number of patients with a measurable PSA at six-month follow-up after surgery was 2 (4%) in both the Da Vinci and Hugo RAS groups, and 4 (8%) in the Versius group, with no statistically significant differences (Supplementary Table 1).

Both minor and major complications and postoperative sequelae occurring beyond 90 days after surgery are detailed in Table 1, stratified by platform. All patients completed the SF-36 and UCLA-PCI questionnaires at baseline (response rate 100%).

**Table 1:** Details of complications, sequelae, and management

Event	Diagnosis time (postoperative days)	Management
**Da Vinci** ^®^ No. events = 17		
1. Incisional hernia 2. Edema of the lower limbs 3. Desaturation (90% SpO_2_) 4. Leakage from the vesicourethral anastomosis (4x) 5. Orchiepididymitis (2x) 6. Myocardial Infarction	*≤* 30 days	1. No current management; hernia repair may be considered if needed. 2. Wearing compression stockings 3. Chest X-ray performed, and antibiotic therapy initiated 4. Maintenance/repositioning of the urinary catheter 5. antibiotic therapy initiated 6. PTCA
1. Orchiepididymitis 2. Incisional hernia (2x) 3. Fluid collection at the midline wound 4. Leakage from the vesicourethral anastomosis (2x)	> 30, *≤* 90 days	1. antibiotic therapy initiated 2. No current management; hernia repair may be considered if needed. 3. Antibiotic therapy initiated 4. Repositioning of the urinary catheter
1. Urethral stenosis with retention	> 90, *≤* 180 days	1. Placement of suprapubic cystostomy and urethrotomy
**Hugo RAS™** **No. events = 18**		
1. Urinary retention 2. Peritoneal effusion 3. Pulmonary embolism originating from thrombosis of the left common and deep femoral venous axis 4. Urinary Tract Infection 5. Bilateral orchitis 6. Leakage from the vesicourethral anastomosis with catheterization 7. Abdominal hemorrhage 8. Pulmonary embolism with intensive care unit admission	*≤* 30 days	1. Catheterization, antibiotic therapy, and cystography prior to catheter removal
1. Penile tumescence 2. Venous ooze from the wound edge 3. Umbilical hernia (2x) 4. Difficulty with adduction of the left thigh 5. Pulmonary embolism originating from thrombosis of the left deep femoral and common femoral veins 6. Recurrent urinary retention	> 30, *≤* 90 days	1. Conservative management, with spontaneous resolution 2. Suture placed after local anesthetic spray 3. No current management; hernia repair may be considered if needed. 4. Electromyography performed: finding of chronic axonal injury of the left obturator nerve: on neurological indication, physiotherapy initiated. 5. Rivaroxaban initiated 6. Cystography and flexible urethrocystoscopy performed; catheter replaced
1. Lymphocele 2. Lymphedema of the right lower limb, involving the inguinal region 3. Recurrent urinary retention	> 90, *≤* 180 days	1. Conservative management 2. Lymphatic drainage massages performed 3. Cystography and flexible urethrocystoscopy performed; catheter replaced
**Versius** ^®^ Number of events = 24		
1. Urinary tract infection 2. Hematuria and pelvic pain in the absence of fever; tenderness at the perineum and right hemiscrotum; right epididymis slightly tender, likely reflecting inflammatory sequelae 3. Findings of scrotal edema, pollakiuria, and dysuria 4. Leakage from the vesicourethral anastomosis (2x) 5. Abdominal pain 6. Urosepsis 7. Fever (urine and blood cultures negative) 8. Abdominal distension (globular, non-tender abdomen), mild tenderness in the right flank. 9. Scrotal edema 10. Initial lymphedema of the right lower limb, involving the inguinal region 11. Pain localized to the perineum in the seated position	*≤* 30 days	1. Antibiotic therapy initiated 2. Started dietary supplement containing bromelain, papain, and fungal proteases 3. Urine culture showed no growth. Conservative management 4. Maintenance/repositioning of the urinary catheter 5. Analgesics initiated 6. Antibiotic therapy initiated 7. Antibiotic therapy initiated 8. Conservative management 9. Conservative management, with spontaneous resolution 10. Lymphatic drainage massages performed 11. Local ice therapy applied
1. Urinary tract infection (3x) 2. Penile tumescence 3. Persistent pain symptoms at the level of the coccyx, perineal area, and penile shaft. 4. Dysuria 5. Lower urinary tract symptoms with weak stream, urinary urgency, and urge incontinence 6. Lymphocele 7. Severe erectile dysfunction	> 30, *≤* 90 days	1. Antibiotic therapy initiated 2. Conservative management, with spontaneous resolution 3. Coccygeal massages of limited success; use of analgesic 4. Cystography and flexible urethrocystoscopy with diagnosis of anastomosis stricture 5. Negative urine culture. Urodynamic diagnosis of hypocontractile and overactive bladder 6. Conservative management 7. penile doppler ultrasound: hemodynamic parameters consistent with erectile dysfunction due to veno-occlusive dysfunction
1. Acute urinary retention associated with pain and vomiting 2. Dysuria 3. Lymphedema of the right lower limb	> 90, *≤* 180 days	1. Placement of suprapubic cystostomy 2. Endoscopic management of a diagnosed stenosis of the vesicoureteral anastomosis. 3. Wearing compression stockings

Supplementary Table 2 includes the questionnaire scores at baseline, derived from the patients’ raw responses, before applying the statistical model.

For Da Vinci, Hugo RAS, and Versius, the response rates were 100, 92, and 98% at 1-month follow-up, 98, 98, and 94% at 3 months, and 100, 94, and 94% at the 6-month monitoring visit, respectively.

The effects of the type of robot used (Da Vinci, Hugo RAS, Versius) on the 0–100 response scores of the SF-36 and UCLA-PCI questionnaires at different study time points (baseline/surgery time, follow-up at 1, 3, and 6 months) were analyzed using a mixed model with robot × time interaction (detailed in Supplementary Table 3). Comparisons between robotic groups at each time point did not show statistically significant differences for most domains analyzed (*p* > 0.05). However, the interaction analysis revealed some significant effects: on the UCLA-PCI questionnaire, the Hugo RAS group showed a mean difference of − 19.73 points (*p* = 0.03, 95% CI [–37.53; − 1.93]), indicating a significant reduction in the Sexual Function domain score compared with the Da Vinci group. The Versius group also showed a significant mean decrease of − 27.7 points (*p* = 0.001, 95% CI [–43.51; − 11.88]) in the Sexual Function score compared with the Da Vinci group at 1-month post-surgery. Finally, again at 1 month postoperatively, the Versius group showed a mean difference of − 25.16 points (*p* = 0.026, 95% CI [–47.35; − 2.97]), suggesting a significant reduction in the Sexual Bother domain score compared with the Da Vinci group.

**Fig. 1:**
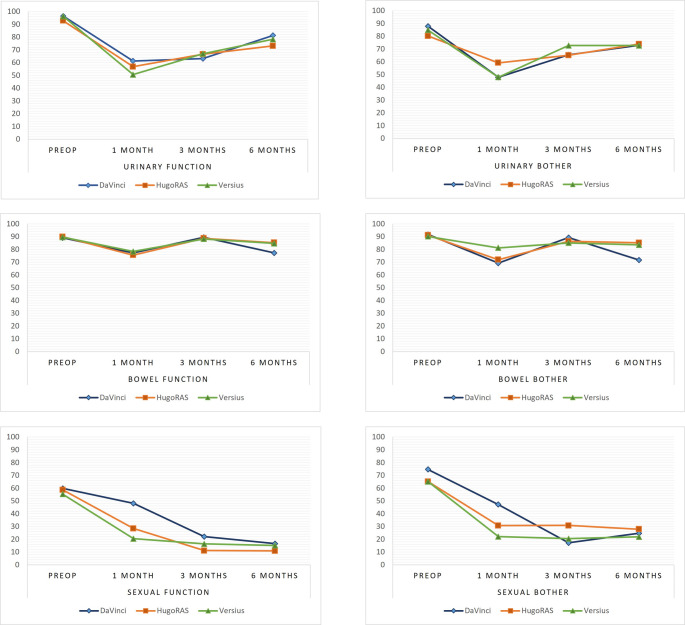
Estimated and adjusted UCLA-PCI mean scores for the six domains before RARP and at each follow-up time point by robotic platform

**Fig. 2:**
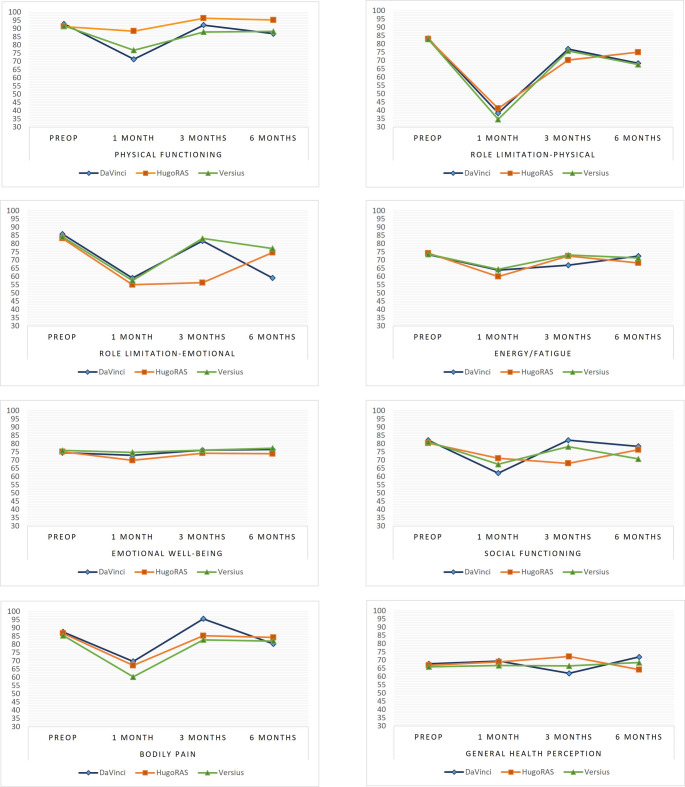
Estimated and adjusted SF-36 mean scores for the eight domains before RARP and at each follow-up time point by robotic platform

As for the SF-36 questionnaire, the only significant difference was observed in the Physical Functioning domain, where Hugo RAS, at 1-month post-surgery, showed an average score of + 17.10 points higher compared with the Da Vinci group (Graphical representations are shown in Figs. 1 and 2).

## Discussion

With the growing diversification of the global surgical robotics ecosystem, multiple platforms have recently entered the urological field, including Hugo RAS, Versius, Senhance, Hinotori, Revo-I, Avatera, KangDuo, Dexter, and Toumai [4]. This raises the question of whether these systems differ in performance and cost.

Several comparative studies have sought to address this issue, though the available evidence remains largely based on initial experiences and heterogeneous study designs.

Some systematic reviews have attempted to synthesize the data, but the significant heterogeneity limits the strength of their conclusions [2–4, 17]. These reviews suggest that the platforms are broadly comparable in terms of safety, functional outcomes, and oncologic efficacy in cancer surgery.

To our knowledge, ours is the first prospective study to compare robotic prostatectomy across three platforms: Da Vinci Xi, Hugo RAS, and Versius.

In the context of an oncological surgical intervention, such as RARP, it is reassuring that—despite the relatively small sample size and limited follow-up—no significant differences were observed in postoperative PSA between patients operated on with different robotic platforms. Six months after surgery, most patients had undetectable PSA levels, regardless of the platform used, suggesting that the oncological safety of the procedure is maintained across platforms.

Most complications were minor and captured within the hyper-controlled trial setting. Interesting in the present work is the description of late postoperative sequelae, recorded between 90 days and the 6-month follow-up visit. These events were anecdotal in nature and comparable across platforms.

Perhaps the most relevant findings come from the analysis of patient-reported outcomes. One of the main strengths of this prospective study lies in the granularity of adverse event reporting, both in the early and short term, but especially in the systematic collection of validated questionnaires. While radical prostatectomy is effective for cancer control, it is often associated with quality-of-life issues related to erectile dysfunction and urinary incontinence. Therefore, integrating patient-reported outcomes into clinical care is crucial to ensure comprehensive and effective treatment [18].

The response rate in our investigation was remarkably high, exceeding 90% and often approaching 100% across all evaluated time points, providing robustness to the results.

From a global perspective, comparisons between robotic systems did not reveal significant differences in most questionnaire domains. However, some differences emerged when focusing on specific outcomes at the 1-month time point. On the UCLA-PCI questionnaire, the Hugo RAS (–20 points) and the Versius platform (–28 points) showed a significant reduction in the Sexual Function domain compared with the Da Vinci platform. In the Sexual Bother domain, Versius also performed worse than Da Vinci (–25 points). The fact that Versius performed worse in the immediate postoperative period in terms of perceived sexual function contrasts, to some extent, with the high rate of positive surgical margins (40%), with a p-value approaching significance not in comparison with Da Vinci, but rather with Hugo RAS. Although baseline disease characteristics did not differ between groups, and nerve-sparing techniques were applied with comparable frequency across platforms, there may be an intrinsic aspect of the console vision that influences dissection. While this cannot be stated with certainty based on our data, given that these observations arise from an exploratory, likely underpowered analysis, it remains a finding that should not be overlooked. That said, this difference in margin status did not translate into variations in the proportion of patients achieving undetectable PSA during follow-up. On the other hand, it is essential to underline that these differences were limited to the 1-month follow-up and disappeared at subsequent 3- and 6-month evaluations, suggesting only a transient early effect.

On the SF-36 questionnaire, the only significant difference was observed in the Physical Functioning domain, where Hugo RAS patients scored on average 17 points higher than Da Vinci patients at 1 month. While this result is more difficult to interpret, one possible explanation is differences in ergonomics, port placement, or convalescence dynamics, which could have temporarily affected patients’ perceived ability to perform daily activities. We cannot comment further on this. Once again, however, these differences were resolved at later follow-ups, reinforcing the notion of equivalence, at least in the short term. Moreover, although some estimates showed numerically potentially relevant differences, the wide confidence intervals suggest that the study may be underpowered to detect significant differences. Further analyses with larger samples may be needed to confirm these trends.

Overall, compared with previously published studies that have focused mainly on perioperative factors such as docking time or operative duration [2–4, 19], our study provides a broader, more detailed picture. Data collection was prospective, with balanced cohorts and without patient selection, and follow-up was particularly rigorous, extending to 6 months.

On the other hand, although patients were unselected and allocated to one of the tested platforms until enrollment of 50 consecutive cases per cohort was completed, the non-randomized nature of the study may still introduce unmeasured selection bias.

The findings support the conclusion that robotic platforms are equivalent in terms of both safety (comparable complication profiles) and oncological efficacy (as indicated by undetectable PSA rates). Ultimately, our results reinforce the notion that surgical outcomes depend less on the type of robotic system used and more on the surgeon’s expertise and technique [20]. Notably, the procedures in this study were performed by high-volume robotic surgeons with extensive Da Vinci experience, who nonetheless achieved comparable results already within their first 50 cases using the alternative platforms [21]. While this prior experience with the Da Vinci system, combined with the lack of prior exposure to Hugo RAS or Versius, may have acted as a confounding factor in early outcomes and reflected an inherent learning-curve effect, this limitation is common to comparative studies of emerging robotic platforms. That said, we believe our findings are highly relevant to real-world practice, as they mirror the typical scenario of experienced Da Vinci surgeons transitioning to alternative robotic systems.

That said, the monocentric nature of the study and the specialized expertise of the surgeons involved may limit the external validity of our findings. In particular, these results may not be directly applicable to settings with varying levels of surgical proficiency or experience, as introducing a novel robotic platform in robotic-naïve centers may pose additional challenges.

Among the multitude of data analyzed, the only signal suggesting a potential “inferiority” of Hugo RAS and Versius concerned early patient-reported sexual function. However, given that these differences were limited to the first postoperative month and no longer evident by 3 and 6 months, their clinical significance remains debatable. A plausible interpretation is that the Da Vinci platform may initially allow more refined nerve-sparing, possibly because surgeons are more familiar with its instrumentation and energy settings, thereby reducing the risk of injury to the neurovascular bundles—although this remains only a hypothesis. Over time, recovery mitigates these differences.

In summary, within the limitations of a relatively limited sample size, the learning curve associated with the surgeons involved, and a short follow-up, this prospective study suggests that different robotic platforms can achieve comparable oncological efficacy, safety, and short-term patient-reported outcomes.

The transient differences observed in some functional domains at 1 month should be interpreted with caution, and further long-term, multicenter data will be valuable for confirming these findings. Given the feasibility-oriented design of the study, perioperative complications were the only prespecified primary outcomes, whereas all other endpoints were analyzed in an exploratory fashion. Accordingly, the study was not powered for formal multiplicity-adjusted hypothesis testing, and any isolated statistically significant findings—particularly at early time points should be interpreted as hypothesis-generating rather than confirmatory.

## Supplementary Information

Below is the link to the electronic supplementary material.

Supplementary Material 1

Supplementary Material 2

Supplementary Material 3

Supplementary Material 4

## Data Availability

All data supporting the findings of this study are available from the corresponding author upon reasonable request.
