# Supplementary material for: Six-month outcomes of a three-arm prospective study comparing Da Vinci vs. Hugo RAS vs. versius robotic radical prostatectomy: (the COMPAR-P trial)
Source: J Robot Surg. 2026 Mar 19;20(1):361. doi: 10.1007/s11701-026-03260-5 (PMC12999588; doi:10.1007/s11701-026-03260-5)
Supplement: Supplementary file 1 — Supplementary Material 1 [file 11701_2026_3260_MOESM1_ESM.docx]

| BASELINE |  | **Da Vinci^®^**  **N=50** | **Hugo RAS^®^**  **N=50** | **Versius^®^**  **N=50** | DV-HR  DV-V  HR-V |
| --- | --- | --- | --- | --- | --- |
|  | Items | Median [IQR] | |  | p-value |
| ***SF-36 questionnaire* (score)**  1. Physical Functioning  2. Role Limitations-Physical  3. Role Limitations-Emotional  4. Energy/Fatigue  5. Emotional Well-Being  6. Social Functioning  7. Bodily Pain  8. General Health Perception | 10  4  3  4  5  2  2  5 | 100 [90 – 100]  100 [100 – 100]  100 [67 – 100]  75 [70 – 80]  76 [64 – 84]  87.5 [75 – 100]  100 [74 – 100]  72 [62 – 82] | 95 [90 - 100]  100 [75 - 100]  100 [67 – 100]  77.5 [70 – 85]  80 [68 - 88]  87.5 [75 - 100]  100 [74 – 100]  72 [57 - 77] | 95 [95 - 100]  100 [75 - 100]  100 [67 -100]  75 [65 - 85]  80 [64 - 92]  87.5 [75 – 100]  100 [74 – 100]  64.5 [52 - 80] | 0.8  0.5  0.3  0.4  0.8  0.6  1  0.8  0.8    0.6  0.8  0.9  0.2  0.3  0.9    0.9  0.5  0.5  0.1  0.2  0.8  0.6  0.2  0.3 |
| BASELINE |  | **Da Vinci^®^**  **N=50** | **Hugo RAS^®^**  **N=50** | **Versius^®^**  **N=50** |  |
|  | Items | Median [IQR] | |  | p-value |
| ***UCLA-PCI questionnaire* (score)**  1. Urinary function  2. Urinary bother  3. Bowel function  4. Bowel bother  5. Sexual function  6. Sexual bother | 5  1  4  1  8  1 | 100 [95 – 100]  100 [75 – 100]  93.8 [85.4 – 100]  100 [100 – 100]  58 [30 – 81]  75 [50 – 100] | 100 [88 - 100]  100 [75 – 100]  93.8 [85.4 - 100]  100 [100 – 100]  63 [42 – 78]  75 [50 – 100] | 100 [100 - 100]  100 [75 – 100]  93.8 [80.4 - 100]  100 [75 – 100]  58 [41 – 72]  75 [38 – 100] | 0.4  0.3  0.1  0.1  0.4  0.5  0.7  0.3  0.5  0.9  0.1  0.2  0.8  0.6  0.4  0.6  0.8  0.8 |

**Supplementary Table 2**. Observed SF-36 and UCLA-PCI median (IQR) outcomes across DaVinci, Hugo RAS, and Versius groups at baseline.
