# Supplementary material for: Six-month outcomes of a three-arm prospective study comparing Da Vinci vs. Hugo RAS vs. versius robotic radical prostatectomy: (the COMPAR-P trial)
Source: J Robot Surg. 2026 Mar 19;20(1):361. doi: 10.1007/s11701-026-03260-5 (PMC12999588; doi:10.1007/s11701-026-03260-5)
Supplement: Supplementary file 2 — Supplementary Material 2 [file 11701_2026_3260_MOESM2_ESM.docx]

**Supplementary Table 1.** Outcomes of Interest

|  | Da Vinci^®^  50 | Hugo RAS^TM^  50 | Versius^®^  50 | p-value  DV\|HR  DV\|V  HR\|V |
| --- | --- | --- | --- | --- |
| *Nerve-sparing surgery*  No. (%) | 12 (24) | 10 (20) | 14 (28) | 0.6  0.9  0.6 |
|  |  |  |  |  |
| *Prostatectomy Gleason Score*  No. (%)  6  7  3+4  4+3  8  9 | 8 (16)  31 (62)  16  15  8 (16)  3 (6) | 6 (12)  29 (58)  15  14  13 (26)  2 (4) | 3 (6)  42 (84)  24  18  4 (8)  1 (2) | 0.4  0.7  0.1 |
|  |  |  |  |  |
| *pT*  *T2a*  *T2b*  *T2c*  *T3a*  *T3b* | 14 (28)  2 (4)  19 (38)  10 (20)  5 (10) | 7 (14)  3 (6)  30 (60)  6 (12)  4 (8) | 16 (32)  3 (6)  21 (42)  9 (18)  1 (2) | 0.8  0.3  0.1 |
|  |  |  |  |  |
| *Lymph-Node Dissection*  No. (%) | 23 (46) | 26 (52) | 16 (32) | 0.5  0.1  <0.05 |
|  |  |  |  |  |
| *No. lymph-nodes dissected*  Median [IQR] | 24  [18 - 26] | 20  [14 - 22] | 14  [9 - 19] | 0.3  <0.05  0.08 |
|  |  |  |  |  |
| *Positive Surgical Margins*  No. (%) | 13 (26) | 11 (22) | 20 (40) | 0.6  0.1  0.05 |
|  |  |  |  |  |
| *Detectable PSA* (≥0.1 ng/mL)  *1 month follow-up*  *Detectable PSA* (≥0.1 ng/mL)  *6 months follow-up*  No. (%) | 5 (10)  _N=50_  2 (4)  _N=50_ | 9 (19)  _N=48_  2 (4)  _N=45_ | 4 (8)  _N=49_  4 (8)  _N=48_ | 0.2  0.7  0.1  0.9  0.4  0.4 |

|  |  |  |  |  |
| --- | --- | --- | --- | --- |
| *>3 pads per day**  *1 month follow-up*  *3 months follow-up*  *6 months follow-up*  No. (%) | 9 (18)  _N=50_  5 (10.2)  _N=49_  4 (8.2)  _N=49_ | 11 (23.9)  _N=46_  6 (12.5)  _N=48_  3 (6.5)  _N=46_ | 9 (19.1)  _N=47_  4 (8.5)  _N=47_  1 (2.1)  _N=47_ | 0.6  0.7  0.5  0.7  0.8  0.5  0.8  0.2  0.3 |

**from UCLA-PCI* *questionnaire:* ***“****How many pads or adult diapers per day did you usually use to control leakage*

*during the last 4 weeks?”*
