# Supplementary material for: Six-month outcomes of a three-arm prospective study comparing Da Vinci vs. Hugo RAS vs. versius robotic radical prostatectomy: (the COMPAR-P trial)
Source: J Robot Surg. 2026 Mar 19;20(1):361. doi: 10.1007/s11701-026-03260-5 (PMC12999588; doi:10.1007/s11701-026-03260-5)
Supplement: Supplementary file 3 — Supplementary Material 3 [file 11701_2026_3260_MOESM3_ESM.docx]

UCLA.PCI questionnaire

| **Domain** | **URINARY FUNCTION** | | | | |
| --- | --- | --- | --- | --- | --- |
| **Time (month)** | **Contrast** | **Estimate mean diff.** | **Std. Err.** | **p** | **95% CI** |
| 0 | H vs DV | -3.66 | 8.52 | 0.667 | [-20.35 ; 13.03] |
| 1 | H vs DV | -4.51 | 8.59 | 0.600 | [-21.35 ; 12.33] |
| 3 | H vs DV | +3.42 | 8.70 | 0.694 | [-13.63 ; 20.47] |
| 6 | H vs DV | -8.23 | 8.59 | 0.338 | [-25.07 ; 8.61] |
| 0 | V vs DV | -0.12 | 7.50 | 0.987 | [-14.83 ; 14.58] |
| 1 | V vs DV | -10.66 | 7.52 | 0.156 | [-25.40 ; 4.08] |
| 3 | V vs DV | +3.83 | 7.55 | 0.612 | [-10.96 ; 18.62] |
| 6 | V vs DV | -2.99 | 7.58 | 0.693 | [-17.84 ; 11.86] |
| **Domain** | **URINARY BOTHER** | | | | |
| 0 | H vs DV | -7.67 | 11.27 | 0.496 | [-29.75 ; 14.41] |
| 1 | H vs DV | +11.25 | 11.37 | 0.323 | [-11.05 ; 33.54] |
| 3 | H vs DV | -0.34 | 11.52 | 0.977 | [-22.91 ; 22.23] |
| 6 | H vs DV | +0.95 | 11.37 | 0.933 | [-21.34 ; 23.25] |
| 0 | V vs DV | -3.04 | 10.01 | 0.761 | [-22.67 ; 16.58] |
| 1 | V vs DV | +0.05 | 10.03 | 0.996 | [-19.61 ; 19.71] |
| 3 | V vs DV | +2.15 | 9.96 | 0.829 | [-17.38 ; 21.68] |
| 6 | V vs DV | -0.17 | 9.99 | 0.986 | [-19.74 ; 19.40] |
| **Domain** | **BOWEL FUNCTION** | | | | |
| 0 | H vs DV | +0.82 | 5.58 | 0.884 | [-10.12 ; 11.75] |
| 1 | H vs DV | -1.71 | 5.63 | 0.761 | [-12.75 ; 9.32] |
| 3 | H vs DV | -0.74 | 5.70 | 0.897 | [-11.91 ; 10.43] |
| 6 | H vs DV | +8.05 | 5.63 | 0.153 | [-2.98 ; 19.09] |
| 0 | V vs DV | +0.51 | 4.91 | 0.918 | [-9.12 ; 10.13] |
| 1 | V vs DV | +1.12 | 4.92 | 0.819 | [-8.52 ; 10.76] |
| 3 | V vs DV | -1.25 | 4.93 | 0.799 | [-10.92 ; 8.41] |
| 6 | V vs DV | +7.50 | 4.95 | 0.130 | [-2.20 ; 17.20] |
| **Domain** | **BOWEL BOTHER** | | | | |
| 0 | H vs DV | -0.61 | 8.12 | 0.940 | [-16.53 ; 15.30] |
| 1 | H vs DV | +2.70 | 8.19 | 0.742 | [-13.36 ; 18.75] |
| 3 | H vs DV | -2.92 | 8.29 | 0.725 | [-19.17 ; 13.33] |
| 6 | H vs DV | +13.43 | 8.19 | 0.101 | [-2.63 ; 29.49] |
| 0 | V vs DV | -1.57 | 7.21 | 0.827 | [-15.70 ; 12.56] |
| 1 | V vs DV | +11.94 | 7.22 | 0.098 | [-2.21 ; 26.10] |
| 3 | V vs DV | -4.10 | 7.17 | 0.567 | [-18.16 ; 9.96] |
| 6 | V vs DV | +11.79 | 7.20 | 0.101 | [-2.32 ; 25.90] |
| **Domain** | **SEXUAL FUNCTION** | | | | |
| 0 | H vs DV | -1.18 | 9.00 | 0.896 | [-18.82 ; 16.46] |
| 1 | H vs DV | -19.73 | 9.08 | 0.030 * | [-37.53 ; -1.93] |
| 3 | H vs DV | -10.86 | 9.19 | 0.237 | [-28.87 ; 7.16] |
| 6 | H vs DV | -5.58 | 9.08 | 0.539 | [-23.38 ; 12.22] |
| 0 | V vs DV | -4.49 | 7.92 | 0.571 | [-20.01 ; 11.03] |
| 1 | V vs DV | -27.70 | 8.07 | 0.001 ** | [-43.51 ; -11.88] |
| 3 | V vs DV | -5.61 | 8.00 | 0.484 | [-21.29 ; 10.08] |
| 6 | V vs DV | -1.39 | 7.97 | 0.862 | [-17.00 ; 14.23] |
| **Domain** | **SEXUAL BOTHER** | | | | |
| 0 | H vs DV | -9.51 | 12.63 | 0.451 | [-34.27 ; 15.24] |
| 1 | H vs DV | -16.43 | 12.74 | 0.197 | [-41.40 ; 8.55] |
| 3 | H vs DV | +13.62 | 12.90 | 0.291 | [-11.67 ; 38.90] |
| 6 | H vs DV | +3.13 | 12.74 | 0.806 | [-21.84 ; 28.11] |
| 0 | V vs DV | -9.51 | 11.24 | 0.397 | [-31.54 ; 12.52] |
| 1 | V vs DV | -25.16 | 11.32 | 0.026 * | [-47.35 ; -2.97] |
| 3 | V vs DV | +3.27 | 11.23 | 0.771 | [-18.75 ; 25.28] |
| 6 | V vs DV | -2.80 | 11.18 | 0.802 | [-24.71 ; 19.11] |

SF-36 questionnaire

| **Domain** | **PHYSICAL FUNCTIONING** | | | | |
| --- | --- | --- | --- | --- | --- |
| **Time (month)** | **Contrast** | **Estimate mean diff.** | **Std. Err.** | **p** | **95% CI** |
| 0 | H vs DV | -1.63 | 6.29 | 0.796 | [-13.95 ; 10.69] |
| 1 | H vs DV | +17.10 | 6.34 | 0.007 * | [+4.67 ; +29.53] |
| 3 | H vs DV | +4.22 | 6.63 | 0.525 | [-8.77 ; 17.20] |
| 6 | H vs DV | +8.37 | 6.34 | 0.187 | [-4.06 ; 20.79] |
| 0 | V vs DV | -1.15 | 5.53 | 0.835 | [-11.98 ; 9.68] |
| 1 | V vs DV | +5.46 | 5.56 | 0.326 | [-5.44 ; 16.36] |
| 3 | V vs DV | -4.17 | 5.79 | 0.471 | [-15.52 ; 7.18] |
| 6 | V vs DV | +1.38 | 5.57 | 0.805 | [-9.54 ; 12.29] |
| **Domain** | **ROLE LIMITATIONS-PHYSICAL** | | | | |
| 0 | H vs DV | -0.38 | 14.01 | 0.979 | [-27.84 ; 27.09] |
| 1 | H vs DV | +2.83 | 14.14 | 0.841 | [-24.89 ; 30.55] |
| 3 | H vs DV | -6.70 | 14.79 | 0.650 | [-35.68 ; 22.28] |
| 6 | H vs DV | +6.66 | 14.14 | 0.638 | [-21.06 ; 34.37] |
| 0 | V vs DV | -0.47 | 12.35 | 0.970 | [-24.68 ; 23.75] |
| 1 | V vs DV | -3.75 | 12.43 | 0.763 | [-28.11 ; 20.62] |
| 3 | V vs DV | -1.13 | 12.97 | 0.930 | [-26.56 ; 24.29] |
| 6 | V vs DV | -0.72 | 12.40 | 0.953 | [-25.04 ; 23.59] |
| **Domain** | **ROLE LIMITATIONS-EMOTIONAL** | | | | |
| 0 | H vs DV | -2.57 | 14.02 | 0.854 | [-30.05 ; 24.90] |
| 1 | H vs DV | -4.15 | 14.15 | 0.769 | [-31.87 ; 23.58] |
| 3 | H vs DV | -25.41 | 14.78 | 0.086 | [-54.39 ; 3.56] |
| 6 | H vs DV | +15.46 | 14.15 | 0.274 | [-12.27 ; 43.19] |
| 0 | V vs DV | -1.58 | 12.34 | 0.898 | [-25.76 ; 22.60] |
| 1 | V vs DV | -1.54 | 12.44 | 0.901 | [-25.93 ; 22.84] |
| 3 | V vs DV | +1.48 | 12.98 | 0.909 | [-23.96 ; 26.92] |
| 6 | V vs DV | +17.90 | 12.41 | 0.149 | [-6.43 ; 42.23] |
| **Domain** | **ENERGY/FATIGUE** | | | | |
| 0 | H vs DV | +0.78 | 5.64 | 0.890 | [-10.28 ; 11.84] |
| 1 | H vs DV | -3.93 | 5.75 | 0.494 | [-15.21 ; 7.34] |
| 3 | H vs DV | +5.56 | 6.01 | 0.355 | [-6.23 ; 17.35] |
| 6 | H vs DV | -4.16 | 5.69 | 0.465 | [-15.32 ; 6.99] |
| 0 | V vs DV | +0.18 | 4.96 | 0.971 | [-9.55 ; 9.91] |
| 1 | V vs DV | +0.47 | 4.98 | 0.925 | [-9.30 ; 10.23] |
| 3 | V vs DV | +6.25 | 5.20 | 0.229 | [-3.94 ; 16.45] |
| 6 | V vs DV | -1.09 | 5.017 | 0.828 | [-10.92 ; 8.74] |
| **Domain** | **EMOTIONAL WELL-BEING** | | | | |
| 0 | H vs DV | +0.59 | 6.49 | 0.927 | [-12.12 ; 13.31] |
| 1 | H vs DV | -3.03 | 6.55 | 0.643 | [-15.87 ; 9.80] |
| 3 | H vs DV | -1.90 | 6.71 | 0.777 | [-15.03 ; 11.23] |
| 6 | H vs DV | -2.63 | 6.55 | 0.688 | [-15.47 ; 10.20] |
| 0 | V vs DV | +1.37 | 5.71 | 0.810 | [-9.81 ; 12.56] |
| 1 | V vs DV | +1.83 | 5.73 | 0.749 | [-9.40 ; 13.07] |
| 3 | V vs DV | -0.01 | 5.73 | 0.998 | [-11.25 ; 11.23] |
| 6 | V vs DV | +0.74 | 5.77 | 0.898 | [-10.57 ; 12.05] |
| **Domain** | **SOCIAL FUNCTIONING** | | | | |
| 0 | H vs DV | -1.78 | 8.59 | 0.836 | [-18.61 ; 15.05] |
| 1 | H vs DV | +9.02 | 8.67 | 0.298 | [-7.96 ; 26.01] |
| 3 | H vs DV | -14.09 | 8.77 | 0.108 | [-31.28 ; 3.09] |
| 6 | H vs DV | -2.08 | 8.67 | 0.810 | [-19.07 ; 14.91] |
| 0 | V vs DV | -0.85 | 7.56 | 0.910 | [-15.66 ; 13.95] |
| 1 | V vs DV | +5.37 | 7.59 | 0.479 | [-9.50 ; 20.24] |
| 3 | V vs DV | -3.94 | 7.60 | 0.604 | [-18.84 ; 10.96] |
| 6 | V vs DV | -7.65 | 7.61 | 0.315 | [-22.56 ; 7.27] |
| **Domain** | **BODILY PAIN** | | | | |
| 0 | H vs DV | -0.88 | 7.58 | 0.908 | [-15.73 ; 13.97] |
| 1 | H vs DV | -2.48 | 7.64 | 0.746 | [-17.46 ; 12.50] |
| 3 | H vs DV | -10.28 | 7.74 | 0.184 | [-25.46 ; 4.90] |
| 6 | H vs DV | +3.88 | 7.64 | 0.612 | [-11.11 ; 18.86] |
| 0 | V vs DV | -2.21 | 6.67 | 0.740 | [-15.28 ; 10.86] |
| 1 | V vs DV | -9.40 | 6.69 | 0.160 | [-22.52 ; 3.71] |
| 3 | V vs DV | -12.77 | 6.71 | 0.057 | [-25.92 ; 0.38] |
| 6 | V vs DV | +1.65 | 6.72 | 0.806 | [-11.53 ; 14.82] |
| **Domain** | **GENERAL HEALTH PERCEPTION** | | | | |
| 0 | H vs DV | -0.86 | 5.22 | 0.869 | [-11.09 ; 9.38] |
| 1 | H vs DV | -0.56 | 5.27 | 0.915 | [-10.89 ; 9.76] |
| 3 | H vs DV | +10.27 | 5.33 | 0.054 | [-0.17 ; 20.72] |
| 6 | H vs DV | -7.71 | 5.27 | 0.143 | [-18.04 ; 2.62] |
| 0 | V vs DV | -1.80 | 4.60 | 0.695 | [-10.82 ; 7.22] |
| 1 | V vs DV | -2.67 | 4.62 | 0.563 | [-11.73 ; 6.38] |
| 3 | V vs DV | +4.52 | 4.62 | 0.328 | [-4.54 ; 13.58] |
| 6 | V vs DV | -3.43 | 4.64 | 0.459 | [-12.52 ; 5.65] |

Time 0 = baseline, before surgery

**Supplementary Table 3.** Estimated mean differences, standard errors (SE), p values, and 95% confidence intervals (95% CI) for fixed factors at each timepoint from mixed model repeated measures analysis adjusted for age at surgery, UCLA-PCI, and SF-36 baseline scores, respectively, serum PSA, and prostate volume.
